# Female genital mutilation in the UK- where are we, where do we go next? Involving communities in setting the research agenda

**DOI:** 10.1186/s40900-018-0103-5

**Published:** 2018-09-17

**Authors:** S. Dixon, K. Agha, F. Ali, L. El-Hindi, B. Kelly, L. Locock, N. Otoo-Oyortey, S. Penny, E. Plugge, L. Hinton

**Affiliations:** 1 Nuffield department of Primary Care Health Sciences, University of Oxford, Oxford, UK; 2 Health Services Research Unit, University of Aberdeen, Aberdeen, UK; 3 Nuffield Department of Clinical Medicine, University of Oxford, Oxford, UK; 4 Nuffield Department of Obstetrics and Gynaecology, University of Oxford, Oxford, UK; 5 Oxford Against Cutting, Oxford, UK; 6 Midaye Somali Development Network, London, UK; 7 FORWARD, London, UK; 8 English Creative Writing Department, Brunel University, London, UK

**Keywords:** Female genital mutilation (FGM), Research and service priorities, Patient and public involvement

## Abstract

**Background:**

Female Genital Mutilation (FGM) is all practices involving cutting, alteration or injury to the female genitalia for non-medical reasons. It is a form of violence against women and children, with no benefits and many harms. In 2014, the UK Government committed to working to eliminate FGM. Steps taken towards this aim included creation of educational and safeguarding resources for professionals, and legislative changes including a mandatory reporting duty for professionals in England and Wales (where if a girl under 18 discloses or is found on examination to have FGM then the professional is mandated to report this to the police), and an FGM Enhanced Dataset applicable to NHS organisations in England requiring the submission of personal data about women and girls who have had FGM to NHS Digital. To date, compliance with dataset returns from primary care services have been low. This report describes using patient and public involvement (PPI) to identify research and service priorities to support communities affected by FGM.

**Methods:**

We held a series of PPI events (4 focus groups, and a multi-agency seminar) in 2015–2016, following the introduction of these legislative changes, speaking to community members, and professionals involved in their care. We asked participants to consider what they identified as research, knowledge and service priorities to support communities affected by FGM.

**Results:**

The impact of these legislative and reporting requirements on the trust needed for community members to seek to consult health services was identified as important for further research. Priorities for service development were holistic services, that met a woman’s needs throughout her lifecourse. Participants emphasised the importance of understanding how to listen, involve and utilise community voices in developing education for professionals, designing services, and developing policy.

**Conclusions:**

There was a desire for change to develop from within affected communities; any learning and resources need to be co-created and constructed in such a way that they can be effectively shared between women, communities, and professionals. Questions remain about how to define community consultation, how to recognise when it was adequate, and how to hear beyond community activists to hear a wider range of voices.

## Plain English summary

Female genital mutilation (FGM) refers to a set of practices involving cutting, alteration or injury to the external female genitalia for non-medical reasons. With no known health benefits, FGM is often associated with immediate and long-term health complications. Over 200 million girls and women worldwide are living with the effects of FGM. Every year 3 million girls and women are at risk of being cut and exposed to harmful health consequences. Globally FGM is concentrated in sub-Saharan African countries from the Atlantic Coast to the Horn of Africa, but women in other countries including Iraq, Oman, Yemen, Indonesia and Malaysia, are also affected. Global migration means FGM is now a worldwide health issue.

In the UK, there is increasing awareness of the importance of FGM as a health and safeguarding issue. There are many communities living in the UK who originate from countries where FGM is practised. Every local authority area is likely to have women and girls who are affected or potentially at risk. FGM has been a crime in the UK since 1985, and the government is committed to eliminating it. Recent legal changes now make it compulsory for doctors, teachers and other regulated professionals to report directly to the police when anyone aged under 18 makes a first hand disclosure of FGM or FGM is found on examination, and for English NHS health organisations to submit data to NHS Digital about women and girls they have seen who have experienced FGM.

Research on the effects of FGM on women and communities in England is limited. In particular, we know little of affected communities’ knowledge and understanding of these new legal changes, and how this might impact on people’s willingness to go to their doctor. Nor do we know how health professionals are responding to these changes.

We held a series of patient and public involvement (PPI) events with women, communities, health professionals, teachers and researchers living and working in England. We wanted to listen and understand more about these issues and what affected communities believe are the healthcare needs and research priorities.

## Background

Female genital mutilation (FGM) refers to a set of practices that involve partial or total removal of the external female genitalia for non-therapeutic reasons [1]. FGM also encompasses practices that involve injury or alteration to the external genitals including gishiri cuts [2], labial stretching and scarification. The procedure has no known health benefits and often puts those affected at immediate and long-term risk of numerous health problems [3]. Immediate physical risks include haemorrhage, pain, shock, infection, urination problems and death [3]. Many affected women go on to endure the consequences for life; they are more likely to have adverse obstetric outcomes [4] and suffer from mental health problems such as depression and PTSD [5].

Over 200 million girls and women worldwide are living with the effects of FGM [6]. Every year, 3 million girls and women are at risk of FGM and exposed to its potentially negative health consequences. Globally FGM is concentrated in a band of sub-Saharan African countries from the Atlantic Coast to the Horn of Africa but women in other countries such as Indonesia are also affected [7].

There is increasing awareness of FGM as an important health issue in the UK. In 2011, an estimated 103,000 women aged 15–49 with FGM were living in England and Wales [8]. The prevalence rate (England and Wales) is estimated to be 4.8 per 1000 population. Whilst there are wide variations - London has the highest, but Manchester, Slough, Bristol, Leicester and Birmingham also have high rates - there are likely to be affected women and girls living in every local authority area [8].

There has been limited research on FGM in England. Africans Unite Against Child Abuse (AFRUCA) explored attitudes towards and experiences of FGM by African communities in Greater Manchester (2015) [9]. They reported a ‘gaping hole’ in terms of education and the provision of support for FGM-affected communities and emphasised the need to involve men in all efforts to end FGM. They described a ‘culture of silence’ where community members would not admit to knowing anyone who would perform FGM, yet ‘everyone knew where to go if they needed a “cutter”’. They found considerable ambivalence about the UK law. Many felt FGM should not be a criminal offence because it is ‘part of their culture’ [9]. These issues of legality may be further compounding the cultural taboo that surrounds discussing and researching FGM. A respondent to the Bristol PEER study into women’s experiences and attitudes to FGM (2010) said ‘traditionally it is very difficult in our country to talk about the affairs of FGM, and it is very shameful to speak on it’ [10]. This shame can influence perceptions of the accessibility of healthcare. The Bristol study concluded that confidence and trust in the health services was minimal. The 2016 PEER study, conducted by FORWARD and Barnardos, captured the views of migrants in Essex and Norfolk and reported that women found it “too hard to open up to their GP” [11].

In addition to difficulty in speaking up, research has hitherto shown communities perceive a lack of awareness of FGM amongst health professionals as well as lack of appropriate clinical services as key areas to be addressed (Bristol PEER) [10]. There have been significant efforts to improve services for FGM-affected women and girls in the UK. For example, including the development of e-learning resources and guidance for health professionals [12, 13]. These are in the context of government efforts to eliminate the practice. At the 2014 Girl Summit, the then UK Prime Minister David Cameron declared a commitment to eliminating FGM, and announced new funding and actions to tackle FGM including guidance for the police, an NHS FGM prevention programme and changes to legislation [14].

FGM has been a specific crime in the UK since the 1985 Prohibition of Female Circumcision Act. This was superseded by the 2003 FGM Act, the scope of which was extended by the Serious Crime Act in 2015 [15]. These amendments add the offence of failure to protect a girl or woman from FGM, provide lifelong anonymity for victims of FGM, and extend the scope of the law extra-territorially. To date, there have been no successful prosecutions for performing FGM in the UK [16]. The 2015 Act also introduced a mandatory reporting duty requiring all regulated health and social care practitioners and teachers in England and Wales to report cases of FGM where there is a first hand disclosure of FGM or FGM is found on examination [17]. There is no available data about the number of cases of FGM reported under this mandatory reporting duty and concerns have been raised about the “failure to evaluate introduction of female genital mutilation mandatory reporting” [16]. We have concerns that the majority of cases of FGM identified through mandatory reporting are historical, and there has been no evaluation on the potential unintended consequences of the mandatory reporting duty on how able women and girls feel to seek help or support for their FGM, both in health and school settings.

Additionally, the UK Health and Social Care Information Centre (HSCIC) (now NHS Digital) have introduced an FGM Enhanced Dataset, making it mandatory for all acute trusts (from July 2015) and GP practices and mental health trusts (from October 2015) in England to submit quarterly data returns detailing FGM-related health data, including patient identifiable information, to the NHS digital enhanced dataset which “supports the Department of Health's NHS Prevention Programme by presenting a national picture of the prevalence of FGM in England” [18]. The HSCIC have published assurances that no individually identifiable data will be published or shared [19]. However, there remains in the specification of this dataset a clause which indicates that data would not be shared unless a “legal or statutory gateway” is created [20]. Future proofing security of such confidential information is a legitimate concern. A recent memorandum of understanding between the Home Office and NHS Digital to enable sharing of confidential information gathered from the NHS to locate and deport undocumented migrants using health services caused concern  [21, 22], although the government have announced that this memorandum will be revised and will no longer be used to seek information about individuals for immigration offences alone [23]. Health professionals remain anxious that however well-intentioned legislation may seem, trust between caregivers and patients will be at risk of irrevocable damage [24–26]. The rate of data submission has been low, notably from primary care. The 2016–2017 annual report shows that only 74 GP practices (out of a total of approximately 7,613 GP practices in England [27]) submitted data returns [28].

In the aftermath of these legislative changes, it is not known what affected communities know and understand about these laws; what their impact is on perceptions of acceptability or uptake of services; whether mandatory reporting could deter young people from speaking up and seeking help, knowing they and their family will be reported to the police; and how these requirements are understood and perceived by professionals, including any impact on their behaviour.

FGM is a harmful practice. Victims need access to services that meet all of their health needs, in all health settings, across the lifecourse. They need to feel able and willing to access those services. Evidence shows these communities are likely to be less able to access health services [29] despite considerable health needs. It is therefore important to explore the impact of recent FGM legislative changes on relationships with health professionals in the context of their wider health. Through the patient and public events we report here, we sought to work with communities and professionals to explore research priorities and understand research and service needs to support both communities and health professionals working with those affected by FGM in the context of these changes.

## Methods

Our aim was to gather voices from English communities affected by FGM and voices from professionals (in healthcare and education) serving those communities. We wanted to listen to what they identify as important research and resource needs and to form a platform from which to co-design research that would be acceptable to participants and useful to people affected by FGM as well as policy makers.

The principle that health and care should be designed to be more person-centred, and that individuals have a right to make decisions about treatments and manage their own health, is now firmly established as policy in many health systems [30–32]. Patient involvement in decisions and organisations has also long been advocated and is actively supported by the National Institute of Health Research in the United Kingdom. This was an exploratory patient and public involvement project (PPI) for which we held a series of focus groups and a seminar [33, 34] that we hope will provide a platform for developing participatory research going forward.

Focus groups were held with women from communities affected by FGM in a large urban centre and smaller multi-cultural city, including an innovative PPI workshop using dramatherapy techniques, and with professionals working with these communities from healthcare and education. The project concluded with an interactive seminar involving representatives from social care, health education, advocacy groups, grassroots organisations and community members.

The focus groups were held in two urban settings. In one centre we held four focus group sessions. We chose focus groups as a methodology because, as a form of group discussion, they are an effective way of exploring people’s views, and explore how and why people think as they do about particular issues [35].

1. FGM survivors, campaigners and advocates (*n* = 11) led by SD. This group included members of a local rights based organisation committed to preventing FGM, including FGM campaigners, and women from Somali, Sudanese, Nigerian, and Gambian communities, including FGM survivors and anti-FGM activists.
2. Health professionals (*n* = 8) including representatives from sexual health, obstetrics and gynaecology, general practice, psychology, and health visitors, led by SD and LH.
3. Teachers (*n* = 4), representing primary and secondary school, led by SD, with one safeguarding lead at a school with high prevalence, and primary school teachers serving relatively high and low prevalence communities
4. In a large urban centre, we held a PPI discussion led by LH and SP with 6 women from Somalia, who earlier in the day had been taking part in a dramatherapy workshop about experiences of FGM

We asked our focus group participants:

i) what aspects of FGM they thought we should research and what questions they thought needed to be asked, or answered,
ii) what would be a useful resource, and how to ensure that resources were useful, believable, relevant and acceptable,
iii) what the future needs were for communities affected by FGM and the professionals working with them,
iv) what barriers and facilitators there were to achieve these aims.

Finally, we held a one-day seminar, as part of the Sheila Kitzinger Programme at Green Templeton College in Oxford, in February 2016. We invited experts from across disciplines, including advocacy groups and survivors working in and around FGM. Participants included members of community groups, (including the Oxford Rose community (an Oxford survivors network), BK Luwo (United women’s organisation based in Oxford) and Midaye Somali Development Network (A grassroots Somali charity based in London), people working in clinical services (including obstetrics and gynaecology, midwifery, health visitors, paediatrics and safeguarding, primary care, psychiatry and sexual health), multi-agency team workers (including police, social services and community outreach workers), legal and ethics experts, members of charitable organisations leading the way in developing understanding of FGM and in providing services and support for women, families and communities (including FORWARD, AFRUCA, Oxford Against Cutting, Shifting Sands, 28 Too Many), academics and researchers working in FGM (from disciplines including creative writing, public health, anthropology, the Refugee Studies Centre Oxford and three recipients of Mary Seacole awards working on FGM related projects). During this meeting, we reflected upon the progress made in working to support communities affected by FGM and eliminate the practice, and so asked all participants to consider and discuss what research and service uncertainties and priorities could include to continue to support progress towards this aim [36].

This article has been written in collaboration with the voices who contributed to our PPI activities. It reflects what we heard during the PPI events, and has been written together with community members, campaigners, and professionals working with FGM affected communities.

### Analysis

The outputs of the focus groups and seminar were collated and summarised thematically [37].

## Results

We were overwhelmed by the passion and energy that participants brought to these PPI events. The willingness to speak out with courage and commitment was noteworthy.

Many felt their culture was stigmatised by the association with FGM, and expressed a wish for a more balanced representation of their cultures and communities, including the positive values and traditions associated with the transition to womanhood. FGM is but one part of women’s lifecourse, and only one thing of many that had happened to them. Women told us that they did not want to feel defined by their FGM, nor be denied the opportunity to consider other health and social issues which may be more important to them.

### What should be researched?

#### Understanding differences

Different generations within diasporan communities may have different health and educational needs with regard to FGM. More understanding of these differences is vital. This included understanding women’s needs related to FGM at different life stages, but also that the needs of those born in the UK may differ from those who had travelled to live in the UK after birth. There is a need to understand diversity both within and between communities and the different information and support needs they may have.

#### How much, where and when?

The need to know more about how much/whether/where/how and why FGM was being performed in the UK now, or by UK residents, was identified as an important knowledge gap that could help inform both service design, preventative work and resource development.

#### Cultural authenticity

Women and professionals discussed that FGM affects a wide range of different communities, with different traditions, FGM practices, and potentially different on-going health consequences. Learning from other groups’ traditions and experiences was identified as necessary and useful, but how and whether resources and stories can or should be shared between cultures was identified as an uncertainty. Having information and resources relevant to all cultures and covering all types of FGM was felt to be a priority, but whether each community needed a personalised resource, or whether unified resources shared across communities was felt to be unknown.

#### Legal changes

The potential impact of the UK legislation, specifically the 2015 changes including mandatory reporting, the FGM enhanced dataset, and the introduction of the offence of failure to protect a child from FGM, was a key area identified that was felt to warrant further exploration. We heard that it would be important to understand what community members know and understand about the legislation. Questions were raised about what community consultation is for, what makes it adequate and how it is fed back to communities, including understanding how community voices were used in policy development. Whether the laws would alter community or community members’ behaviour, their perception of the accessibility of health services, or influence how professionals interacted with families from affected communities was identified as an important area for further research.

### What services do women want?

Women asked us to consider their health needs throughout their life course, stretching from puberty, to their needs before and during marriage, pregnancy and childbirth, and through to the menopause. They noted that antenatal care is an important time to engage with women about FGM, but that they wanted services that looked after women both through the antenatal period and beyond. They asked for services to not be solely focussed on sexual and reproductive health.

While the term “mental health” was identified as potentially difficult by some community members, describing a perception of stigma against mental illness, (we were told that in many cultures the word “mental” is heavily aligned with “being mad” in common parlance), participants requested holistic service provision, in which both physical and emotional or psychological needs were able to be met. The impact of FGM on both women’s and men’s psycho-sexual function was raised as important to learn more about, including how to support couples affected by FGM. The need for safe spaces to discuss FGM, in the context of known difficulties, and pre-existing cultural taboo’s around discussing FGM was raised, and there was considerable reflection on what these might look like.

The appropriate *setting* for FGM support services was discussed as something that needed exploring, including whether health was the right location for this, and if so whether hospital or community settings would be more acceptable. Many participants questioned whether health settings were the best or only place to discuss FGM. In particular, some uncertainty was raised in one group about whether GPs are the right people to be asking about FGM.

The need for professionals to be aware of FGM, including having skills to respond appropriately to women with FGM without seeming to be horrified or judgemental, was identified as important, with women describing their distress at reactions they or their friends had experienced in healthcare encounters.

### What future needs were identified?

The best way of educating professionals was discussed, including learning how to incorporate community voices and experience into the education offered to professionals. Learning how to continue to break down barriers to talking about FGM, for community members and professionals was identified as important. The involvement of affected communities in *designing* services and facilitating acceptability and usage of services was a recurrent theme, and the role of community facilitators, or health advocates, both to support community members when accessing services, but also in training and educating professionals and developing services was felt to be important to learn more about, and consider implementing. The need to learn more about how legislation may be altering the types of FGM being practiced now, and how much FGM was occurring, when and where, was important.

### What are the barriers

#### Legislation

The potential impact of the new legislation, on whether services could still be considered as safe and confidential, or acceptable to community members, was a dominant theme in all of our focus groups. The potential impact of the requirement to record and to report FGM to authorities without the consent of the community member on trust was consistently voiced as a major concern throughout this work, by community members (activists, advocates and community members) and by professionals (across health, education, and social care). Specific concerns were also raised by both community members and health professionals about a perceived double standard in the law, specifically, the FGM Act 2003- in particular that FGM is illegal yet there is a permissive societal attitude towards female cosmetic genital surgery which also comprises a range of genital-altering procedures for non-medical reasons.

Some expressed their views that the legislation against FGM was stigmatising and some participants described feeling that it was discriminatory against them and their culture. The community members we spoke to were united in their understanding that FGM is a form of child abuse, and in their commitment to supporting communities and families to protect their children from FGM (safeguarding). Some women observed however, that the safeguarding procedures for FGM were significantly different from the regulations for other forms of child abuse, and that they perceived this as discriminatory and disproportionate.

#### Cross cultural learning?

Much of the UK research into FGM has been undertaken within single community groups. One of our focus groups, and the multi-agency meeting included participants from a number of community groups (including women and men from Nigeria, Gambia, Ghana, Sudan, and Somalia). There was uncertainty about how transferable the experiences and needs of one community group may be to other communities, who may have different traditions and needs. How and whether to share experiences between communities, and how to create resources was raised. Many felt that learning from different communities’ experiences was informative and enriching, but for health education and resources, there was uncertainty about how this process could be used. There are repeated calls for resources to be community based, but a lack of clear understanding about what this entails, and how we can know if it has been achieved.

#### Whose voices?

The contribution of anti-FGM activists was acknowledged and hugely valued, but we were asked how to challenge the assumption that they speak for whole communities, and to create opportunities from which to hear and add previously unheard voices to the debate around FGM. How to involve men in the campaign against FGM, and also to understand their views and needs regarding FGM was identified as a research priority. We were asked whether anyone had researched the beliefs and needs of the cutters themselves, noting that they may depend on FGM for financial and status reasons, and therefore we should learn more about how to involve them in aspiring to eliminate the practice of FGM. The question of whether “any” FGM, or type of FGM was acceptable was raised.

## Discussion

As far as we are aware, this is the first published patient and public involvement project asking communities affected by FGM, and professionals involved in providing care for these communities, what they identify as research and service priorities. Previous research in England has gathered information about participants lived reality, experience and attitudes and has provided valuable knowledge about participants' experiences of services, and views about service needs and issues including the UK legislation [10, 11]. This study was a patient and public involvement project, in which we sought explicitly to gather community and professional views as part of an exercise in determining research and service priorities. Sharing experiences was not expected or asked for as part of the sessions undertaken. We referred to the INVOLVE principles of developing and designing research [38] with the public and people the research will be relevant to, and to the James Lind principle of patients and clinicians collaborating in setting priorities [39].

We were overwhelmed by all that we heard, and the number and breadth of questions we were asked. Participants had a great deal to say about FGM and there were forthcoming with their knowledge and ideas for the future.

Our contributors highlighted the complexity and diversity of experiences and attitudes towards FGM across the UK. We have been challenged to think more pluralistically about: where, when and how to address FGM during a woman’s lifecourse; where and when services should be addressing FGM; what communities to listen to; and how to listen to them.

This PPI project took place in the aftermath of the introduction of legislative changes around FGM and they were being frequently reported in the UK media; [40, 41]) which may have influenced the topics identified as important. However, we believe there is a significant need to take forwards in research the questions that were raised during this project, for example to consider what impact the legislative changes has had on behaviour and attitudes of community and professionals, and how to understand how to hear and use community voices when developing legislation and policy that affects them.

### Strengths and limitations

This was an initial exploratory project, and was carried out in only two cities in England. Nonetheless, the enthusiasm for engagement in determining research priorities was striking, which we believe demonstrates the feasibility of involving community members in future research. However our work also provides insights into the complexities of work in this area, and the challenges of undertaking research that engages with FGM practices across many different nationalities and the socio-cultural specificity of experiences and priorities in FGM services. Previous studies have focused on communities in isolation from each other [10, 11]. We had experience of one focus group made up of women from one community, and another where the focus group included women from different community backgrounds, as did the multi-agency workshop. While the numbers here are too small to draw conclusions, we reflected about whether and how different communities could or should learn from each others’ experiences, what may be lost from mixing groups and what might be gained by sharing between communities.

In planning future work we are clear that research should be co-produced with the communities that it seeks to understand and serve. These workshops revealed the importance of socio-culturally authentic research in this highly contested area. While it doesn’t provide a roadmap to co-design, it does demonstrate that communities are willing to engage with collaborative approaches to priority setting and research development.

## Conclusion

There was an overarching desire for solutions, resources and change to develop from communities upwards to professionals and authorities. Any learning and resources need to be co-created and constructed in such a way that they can be effectively shared between women, communities, and professionals. This would allow understandings of what will create effective change, services and training. We need to understand how we can develop and support this process happening, and what techniques, resources and research would be needed to allow this. We need to learn this from the communities themselves.

## Abbreviations

FGM: Female genital mutilation
PPI: Patient and public involvement

## References

[1] World Health Organization. Factsheet on female genital mutilation. http://www.who.int/mediacentre/factsheets/fs241/en/.

[2] Blessing Uchenna Mberu “Female genital mutilation/cutting in Nigeria: A scoping review. May 2017,” Evidence to End FGM/C: Research to Help Women Thrive. New York: Population Council. http://www.popcouncil.org/uploads/pdfs/2017RH_FGMC-NigeriaScopingReview.pdf.

[3] World Health Organization (2016). WHO guidelines on the management of health complications from female genital mutilation.

[4] WHO study group on female genital mutilation and obstetric outcome (2006). Female genital mutilation and obstetric outcome: WHO collaborative prospective study in six African countries. Lancet.

[5] Berg RC, Denison E, Fretheim A (2010). Psychological, social and sexual consequences of female genital mutilation/cutting (FGM/C): a systematic review on quantitative studies. Report from Kunnskapssenteret nr 13-2010.

[6] Female genital mutilation/cutting: a global concern. Geneva: UNICEF; 2016. http://data.unicef.org/resources/female-genitalmutilation-cutting-a-global-concern.

[7] Female genital mutilation/cutting: a statistical overview and exploration of the dynamics of change. New York: United Nations Children’s Fund; 2013. http://data.unicef.org/resources/female-genital-mutilation-cutting-a-statisticaloverview-and-exploration-of-the-dynamics-of-change.

[8] Macfarlane A, Dorkenoo E (2015). Prevalence of Female Genital Mutilation in England and Wales: National and local estimates.

[9] Voices of the Community: Exploring Female Genital Mutilation in the African Community across Greater Manchester. AFRUCA; 2015.

[10] Women’s Experiences and Attitudes of Female Genital Mutilation, The Bristol PEER study, Dr Eiman Hussein and FORWARD January 2010.

[11] “Between Two Cultures” a rapid PEER study Exploring Migrant Community’s Views on Female Genital Mutilation in Essex and Norfolk UK, Barnardos and FORWARD March 2016.

[12] FGM e-learning module. http://www.e-lfh.org.uk/programmes/female-genital-mutilation/.

[13] Department of Health (2017). Safeguarding women and girls at risk of FGM - GOV.UK.

[14] PM hosts Girl Summit 2014: a future free from FGM and child and forced marriage. https://www.gov.uk/government/news/pm-hosts-girl-summit-2014-a-future-free-from-fgm-and-child-and-forced-marriage.

[15] Female Genital Mutilation legal guidance, CPS guidance. http://www.cps.gov.uk/legal/d_to_g/female_genital_mutilation/.

[16] Gerry F, Rowland A, Fowles S, et al. Failure to evaluate introduction of female genital mutilation mandatory reporting. Arch Dis Child. 2016; 10.1136/archdischild-2016-311000. 10.1136/archdischild-2016-311000 27281454

[17] Home Office procedural information for mandatory reporting published 20.10.2015. https://assets.publishing.service.gov.uk/government/uploads/system/uploads/attachment_data/file/573782/FGM_Mandatory_Reporting_-_procedural_information_nov16_FINAL.pdf.

[18] HSCIC Female genital mutilation datasets. http://www.hscic.gov.uk/fgm.

[19] Department of Health, London, 2015, FGM Prevention Programme Understanding the FGM Enhanced dataset – updated guidance and clarification to support implementation. September 2015. https://assets.publishing.service.gov.uk/government/uploads/system/uploads/attachment_data/file/461524/FGM_Statement_September_2015.pdf.

[20] Female Genital Mutilation(FGM) Enhanced dataset, requirements specification, SCCI 2026 - FGM Enhanced Dataset - Requirements Specification v1.0 April 2015. https://digital.nhs.uk/data-and-information/information-standards/information-standards-and-data-collections-including-extractions/publications-and-notifications/standards-and-collections/scci2026-female-genital-mutilation-enhanced-dataset.

[21] Gulland A. Handing NHS data to the Home Office. BMJ. 2017;356. 10.1136/bmj.j911. (Published 22 February 2017) 10.1136/bmj.j911 28228374

[22] Hiam L. Grenfell survivors shouldn't be afraid to go to hospital. BMJ. 2017;358. 10.1136/bmj.j3292 28684432

[23] NHS Digital, Memorandum of Understanding revision plan. https://digital.nhs.uk/services/national-back-office-for-the-personal-demographicsservice/memorandum-of-understanding-revision-plan. Accessed 9 Aug 2018.

[24] Bewley S, Kelly B, Darke K, Erskine K, Gerada C, Lohr P, de Zulueta P (2015). Mandatory submission of patient identifiable information to third parties: FGM now, what next?. BMJ.

[25] Mathers N, Rymer J (2015). Mandatory reporting of female genital mutilation by healthcare professionals. Br J Gen Pract.

[26] Dixon S (2015). The FGM enhanced dataset: how are we going to discuss this with our patients?. Br J Gen Pract.

[27] NHS digial dataset. https://digital.nhs.uk/data-and-information/publications/statistical/female-genital-mutilation/female-genital-mutilationfgm-annual-report-2016-17-pas.

[28] BMA background media briefing April 2017

[29] Norman K, Hemmings J, Hussein E, Otoo-Oyortey N. July 2009 FGM is always with us Experiences, Perceptions and Beliefs of Women Affected by Female Genital Mutilation in London Results from a PEER Study. https://www.options.co.uk/sites/default/files/uk_2009_female_genital_mutilation.pdf

[30] Berwick DM (2009). What ‘patient-centered’ should mean: confessions of an extremist. Health Aff (Millwood).

[31] Coulter A (2011). Engaging Patients in Healthcare.

[32] INVOLVE (2015). Public involvement in research: values and principles framework.

[33] Brown LJ, Dickinson T, Smith S, et al. Openness, inclusion and transparency in the practice of public involvement in research: A reflective exercise to develop best practice recommendations. Health Expect. 2017; 10.1111/hex.12609 PMC5867325 29105227

[34] NIHR. Going the extra mile: Improving the nation's health and wellbeing through public involvement in research. https://www.nihr.ac.uk/patients-and-public/documents/Going-the-Extra-Mile.pdf.

[35] Kitzinger J. Qualitative Research: Introducing focus groups. BMJ. 1995;311 10.1136/bmj.311.7000.299. (Published 29 July 1995) 10.1136/bmj.311.7000.299 PMC2550365 7633241

[36] FGM: Where have we got to? And what comes next? Report from the Sheila Kitzinger Programme event, February 2016. http://www.gtc.ox.ac.uk/research-centres/sheila-kitzinger-programme/sheila-kitzinger-programme-events.html. http://www.gtc.ox.ac.uk/images/Report_from_the_Sheila_Kitzinger_seminar.pdf.

[37] Patton MQ (1990). Qualitative Evaluation and Research Methods.

[38] NIHR INVOLVE, what is public involvement in research. http://www.invo.org.uk/find-out-more/what-is-public-involvement-in-research/. Accessed 23 Feb 2018.

[39] The James Lind Alliance. http://www.jla.nihr.ac.uk/about-the-james-lind-alliance/.htm. Accessed 23 Feb 2018.

[40] Law to make reporting of FGM mandatory https://www.bbc.co.uk/news/uk-31448049.

[41] FGM. Legal duty to inform police comes into force https://www.bbc.co.uk/news/health-34681057.

